# Treatment of Acute Orchitis by the Alternate Application of Heat and Cold

**Published:** 1867-05

**Authors:** 


*Middlesex Hospital.—Treatment of Acute Orchitis by the Alternate application of Heat an Cold.* Under the care of Mr. Nunn.

In private practice there is, in the treatment of gonorrhœa, no complication so much dreaded by the surgeon as that of swelled testicle; especially since the occurrence of the disorder of the testicle is too frequently attributed to some fault of management. The severity of the pain and the "sympathetic" disturbance of the general system have doubtless originated the very active plans of treatment often pursued in such cases. Whether surgical pathologists are strictly correct in teaching that the inflammatory action is limited to the epididymis, or not, it can scarcely be denied that the "sympathetic" phenomena have frequently relation to the nerve-supply of the body of the testis itself, or, are such as would result from violent impressions on the body of the testis. The pain of ordinary swelled testicle is very variable. In some cases the distress is great from a terrible weight in the loins; in other cases the pain is chiefly referred to the crural branch of the genito-crural nerve; but in all cases swelling is the remarkable symptom. The swelling greatly depends on effusion into the tunica vaginalis, though of course the highly vascular and abundant connective tissue of the epididymis and the equally vascular structures of the scrotum have also an important share in the general enlargement. Mr. Nunn employs the alternate influence of hot and cold water in overcoming the inflammatory engorgement, having recourse to compression by strapping only in the late stage of the affection, when tenderness has in a great measure subsided.

Case 1. (No. 80 in Register.)—J. H——, aged twenty-six, was admitted on the 30th of May last, under the care of Mr. Nunn. He had swelled testes one week: gonorrhœa three weeks. No sickness nor pain in the loins. Ordered to have a hot bath with cold douche over the testicle daily; half drachm doses of tincture of sesquichloride of iron three times a day.

In two days the symptoms were greatly relieved. His stay in the hospital was twenty-one days, owing to the continuance of the urethral discharge. The testicle was strapped on the 6th of June.

Case 2. (No. 153 in Register.)—W. P——, aged twenty-three, was admitted on the 27th of June last, with acute orchitis and gonorrhœa. He was ordered a hot bath with cold douche daily, and a compound copaiba draught.

On the 4th of July he was discharged cured. The date of attack was June 21st. Stay in hospital seven days.

Case 3. (No. 236 in Register.)—J. H——; age not recorded. Admitted July 26th. Four leeches and a purgative were ordered by the house-surgeon. On the 28th a hot bath with cold douche was ordered twice a day by Mr. Nunn.

The patient was discharged cured on the 2d of August. His stay in the hospital was six days. The testicle was strapped before he was discharged.

The beneficial influence of alternate application of heat and cold probably depends on the removal of the condition of *stasis* by the alternate dilation and contraction of the vessels in or near the inflamed parts. The crowded and adherent blood-corpuscles are again passed into circulation, and nutritional are substituted for inflammatory processes.

The method of applying heat and cold to the testes is as follows:—The patient is placed in a hot bath; after six or ten minutes a current of cold water is directed on to the testicle by means of an india-rubber tube for a minute or two. The parts are again heated, and the application of the cold water is repeated in a similar manner three or four times. An almost immediate sense of relief is the usual result.—*Ib.*

